# Recurrent immunotactoid glomerulopathy in a kidney transplant recipient: Case report

**DOI:** 10.1097/MD.0000000000043673

**Published:** 2025-08-01

**Authors:** Shuxin Yu, Xiaoxuan Zhao, Fanqian Cheng, Chunbo Zhao, Jinyu Yu, Shan Wu, Weixia Sun

**Affiliations:** a Department of Nephrology, The First Hospital of Jilin University, Changchun, Jilin Province, China.

**Keywords:** immunotactoid glomerulopathy (ITG), kidney transplantation, rituximab

## Abstract

**Rationale:**

Immunotactoid glomerulopathy (ITG) is a rare glomerular disease characterized by protein deposition in hollow microtubules on electron microscopy. Patients may present with proteinuria, hematuria, hypertension, and renal insufficiency, and some patients even progress to end-stage renal disease (ESRD). In patients with ESRD, ITG recurs in more than 50% of patients after kidney transplantation; however, there is no clear treatment plan for these patients owing to the limited number of reported cases.

**Patient concerns:**

In this study, we report a case of a 58-year-old male who was admitted to our hospital with elevated blood creatinine with proteinuria.

**Diagnoses:**

Renal biopsy suggested membranoproliferative glomerulonephritis with findings suggestive of ITG on electron microscopy.

**Interventions:**

After the first diagnosis of renal insufficiency, the patient was treated with rituximab; however, the patient’s symptoms did not improve, and blood creatinine continued to increase. The patient progressed to ESRD, and 3 years later, kidney transplantation was performed. After surgery, he was regularly treated with tacrolimus, mycophenolate mofetil, and prednisone acetate for antirejection. Five months after surgery, a renal biopsy was performed again due to proteinuria, and the results suggested ITG recurrence. The patient’s blood CD19-positive B-cell count was 0. Therefore, he was not administered rituximab again.

**Outcomes:**

The patient is now under regular review; his blood creatinine is relatively stable (120–150 mmol/L), while his 24-hour urine protein quantification is higher than the normal range.

**Lessons:**

This study expands the number of reported cases of this condition and will be useful to better understand the treatment options for patients with ITG recurrence after kidney transplantation.

## 1. Introduction

Immunotactoid glomerulopathy (ITG) was first described by Schwartz and Lewis in 1980.^[1]^ ITG is a rare glomerular disease with a renal biopsy detection rate of only 0.06%.^[2]^ ITG can be divided into 2 pathologic types: membranous nephropathy and membranoproliferative glomerulonephritis,^[3]^ which manifest as glomerular Congo red-negative immunoglobulin (Ig) deposits, with hollow microtubule protein deposits on electron microscopy. These features are often necessary to distinguish ITG from other glomerular disorders, such as cryoglobulinemia and fibrillary glomerulonephritis.

Patients with ITG may present with proteinuria, hematuria, hypertension, and renal insufficiency. Some patients also present with hematological malignancies, with chronic lymphocytic leukemia being the most common.^[3]^ ITG can recur in patients who undergo kidney transplantation, and the optimal treatment option for these patients is unknown due to the limited number of reported cases.

In this case report, we present our experience of a patient with ITG who progressed to end-stage renal disease (ESRD) and who required kidney transplantation, after which ITG recurred. We hope our findings add to the existing literature on the characteristics and treatment strategies for patients with ITG recurrence after kidney transplantation for ESRD.

## 2. Case report

A 58-year-old male patient was admitted to our hospital in October 2018. Clinical examination revealed elevated blood creatinine (204 μmol/L) with proteinuria (++++). Blood pressure was 150/90 mm Hg, there was no obvious bilateral lower limb or facial edema, and the estimated glomerular filtration rate was 46 mL/min/1.73 m^2^, calculated based on the blood creatinine concentration. The patient was diagnosed with stage 3 chronic kidney disease and renal hypertension.

The patient had a history of hypertension for 25 years, and blood pressure was maintained at 150/100 mm Hg after combined application of antihypertensive drugs. Other relevant findings at the time of admission were as follows: total protein in the urine, 5.668 g/24 hours; creatinine, 238 μmol/L; anti-glomerular basement membrane antibodies; and antineutrophil antibodies. Immunofixation electrophoresis of the blood and urine was performed, and kappa and lambda free light chains in the blood were measured. Bone puncture, lung computed tomography, and electrocardiogram did not show any significant abnormalities (Table 1). Table 1 clearly demonstrates that the patient already presented with massive proteinuria, elevated serum creatinine, reduced estimated glomerular filtration rate (eGFR), and moderate anemia at the initial clinical evaluation. These findings collectively indicate the presence of significant renal insufficiency.

**Table 1:** Initial laboratory investigation upon admission.

Parameters	Value	References range	Unit
24-h urinary protein	5.668	0–0.2	g
24-h urinary microalbumin	5174	0–30	mg
Serum creatinine	238	57–97	µmol/L
eGFR	25.3	>90	mL/min
Total protein	48.1	65–85	g/L
Serum albumin	32.3	40–55	g/L
White blood cell count	6.99	3.50–9.50	× 10^9^/L
Red blood cell count	2.95	4.30–5.80	× 10^12^/L
Hemoglobin	95	130–175	g/L
Platelet	208	125–350	× 10^9^/L
Cholesterol	5.9	2.6–6.0	mmol/L
Triglyceride	1.29	0.28–1.80	mmol/L
Acid	394	208–428	µmol/L
Erythrocyte sedimentation rate	41	0–15	mm/h
IgG	5.39	7–17.2	g/L
C3	0.753	0.8–2.0	g/L
C4	0.221	0.1–0.4	g/L
IgM	0.79	0.4–2.3	g/L
IgA	1.22	0.7–4.0	g/L

eGFR = estimated glomerular filtration rate, Ig = immunoglobulin.

**Figure 1.**
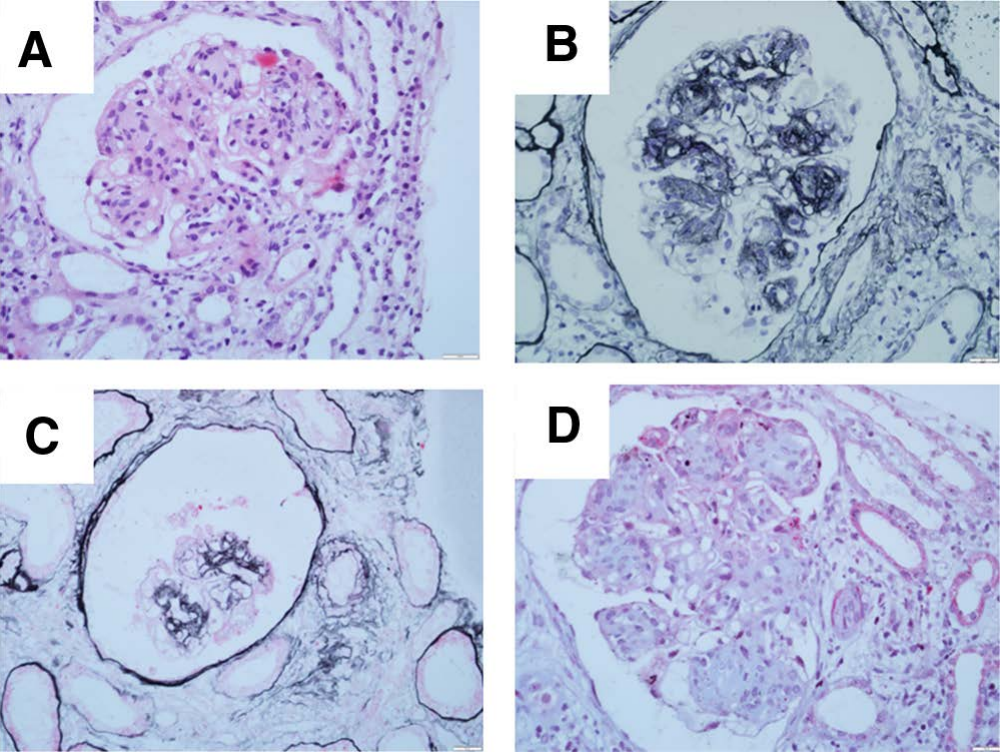
Renal biopsy performed in 2018. (A) H&E staining. (B) Periodic acid-Schiff staining (original magnification × 400) showing diffuse proliferation of glomerular mesangial cells and endothelial cells with increased mesangial stroma. (C) Periodic acid-silver methenamine (original magnification × 400). (D) Masson trichrome (original magnification × 400) showing diffuse hyperplasia with increased mesangial stroma in the glomerular endothelial cells and endothelial cells. Subcutaneous segmental complex red deposition and the double-track sign can be seen.

**Figure 2.**
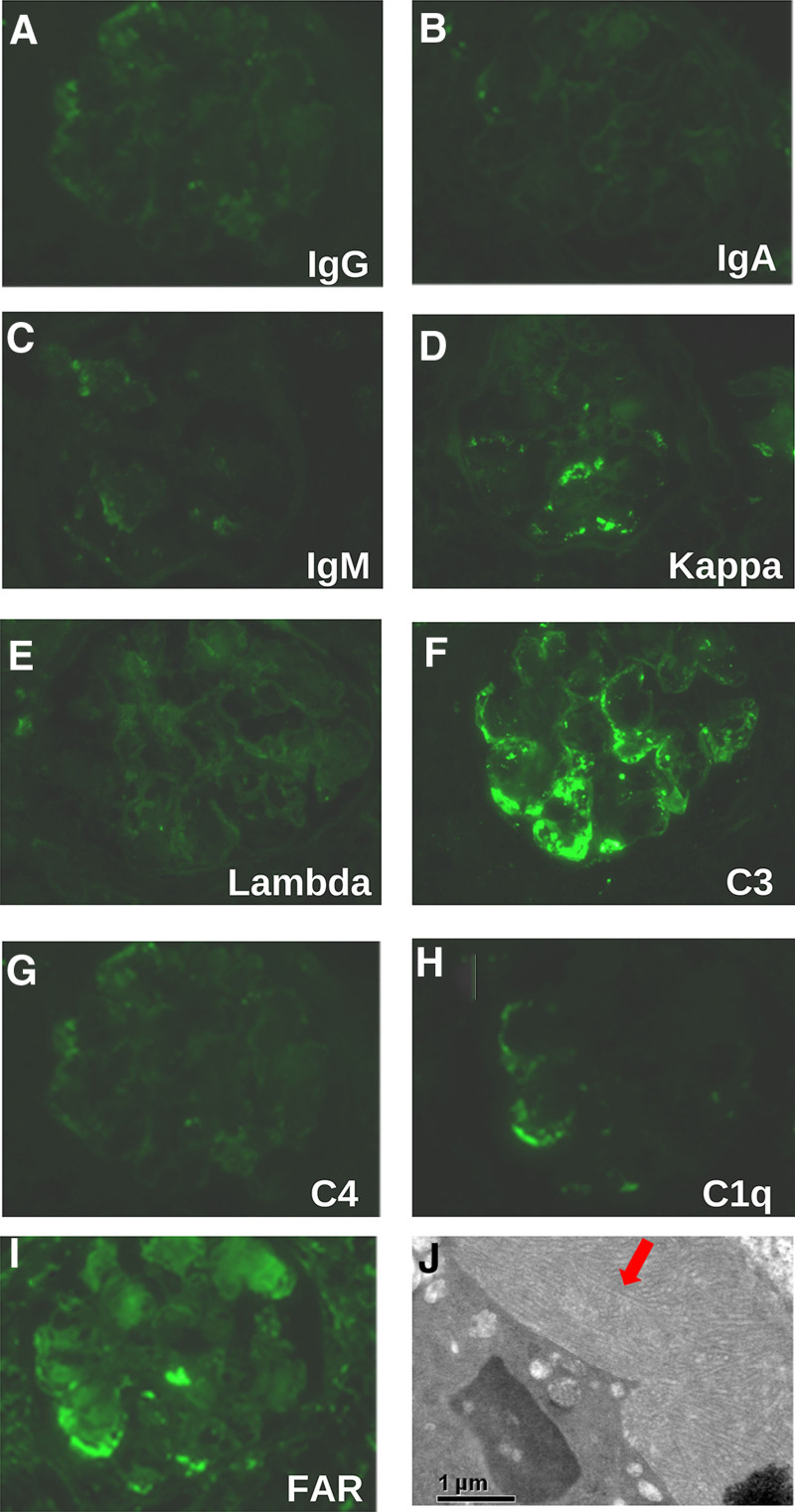
Immunofluorescence microscopy and electron microscopy findings in 2018. Immunofluorescence microscopy (original magnification × 400) showing IgG (+) (A), IgA (+) (B), IgM (++) (C), kappa (+) (D), lambda (+) (E), C3 (+++) (F), C4 (++) (G), Clq (++) (H), and FAR (+) (I). (J) Electron microscopy (original magnification × 15,000) showing a large number of microtubule-like structures in the subendothelial and thylakoid regions. Ig = immunoglobulin (arrows). The images are from the Electron Microscopy Department of Peking University First Hospital.

To clarify the etiology of chronic kidney disease, the patient underwent renal puncture biopsy. Twenty-nine glomeruli were seen in the punctured renal tissue, of which 2 exhibited glomerulosclerosis and 3 exhibited slightly crumpled glomerular filopodia. The balloon lumen was relatively enlarged, and the glomerular mesangial cells and endothelial cells demonstrated diffuse hyperplasia, accompanied by an increase in mesangial stroma. The glomeruli were lobular, with poorly opened capillary vessels, and inflammatory cell infiltration was seen in the segmental glomeruli. Moreover, peripheral adhesion of the segments to the wall of the balloon was accompanied by thickening and layering, and there was pericapsular fibrosis of the segmental balloon wall. PASM-Masson staining showed subglomerular subendothelial deposition of complex red material in the segments. Acute tubulointerstitial lesions were mild-to-moderate, with multiple small foci of tubular epithelial cells with brush border detachment. This was accompanied by mild chronic lesions, small foci of tubular atrophy, basement membrane thickening, a protein pattern in individual tubules, and mild widening of the tubulointerstitial segments. Mild fibrosis with scattered lymphocyte and mononuclear macrophage infiltration was also observed, and there were no obvious lesions in the small arteries (Fig. 1A–D). Immunofluorescence showed IgG (+), IgA (+), IgM (++), kappa (+), lambda (+), C3 (+++), C4 (++), Clq (++), and FAR (+) distribution in the segments (Fig. 2A–I), deposited in a wreath shape and mainly present in vascular collaterals. Electron microscopy suggested a large number of microtubule-like structures in the subendothelial and thylakoid regions (Fig. 2J). Secondary glomerulonephritis caused by diabetes mellitus, autoimmune diseases, solid tumors, hepatitis B virus, hepatitis C virus, and medications was excluded. The clinical diagnosis of ITG was made by combining the patient’s symptoms, signs, clinical presentation, electron microscopy findings, and light microscopy findings.

The patient was treated with 6 rounds of rituximab (100 mg) administered by intravenous injection each time from 2019 to 2021. During the posttreatment review period, blood creatinine gradually increased (Fig. 7A), urine protein status improved compared to the previous period and was maintained at 4 to 7 g/24 hours (Fig. 7B), and blood albumin was < 30 g/L. Kidney transplantation was performed in 2021 due to uremia, and the patient was regularly treated with tacrolimus, mycophenolate mofetil and prednisone acetate after surgery.

**Figure 3.**
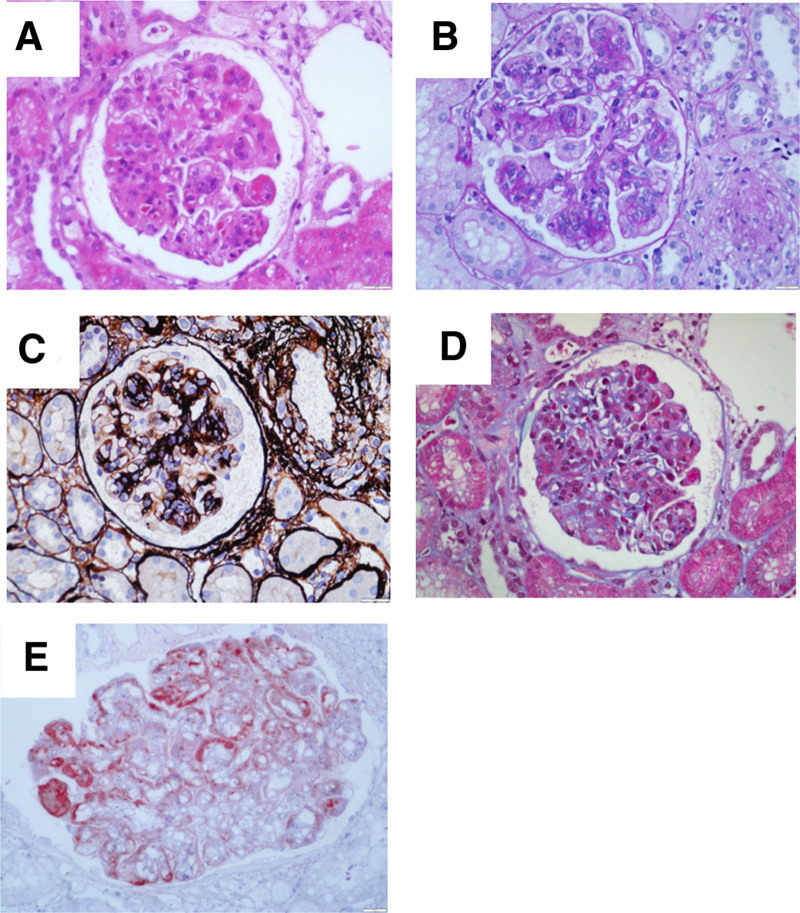
Renal biopsy performed in 2022. (A) H&E staining. (B) Periodic acid-Schiff staining (original magnification × 400). (C) Periodic acid-silver methenamine staining (original magnification × 400). (D) Masson trichrome (original magnification × 400) showing glomerular mesangial cells and thylakoid stroma with diffuse mild-to-moderate hyperplasia, segmental hyperplasia with severe exacerbation, segmental endothelial cell swelling with marked hyperplasia, insertion of hyperplastic thylakoid stroma into the capillary wall, segmental thickening of the glomerular basement membrane, formation of segmental double-track sign, and complex deposition in the subendothelium. (E) C4D staining deposited along the capillary wall. H&E = hematoxylin and eosin.

**Figure 4.**
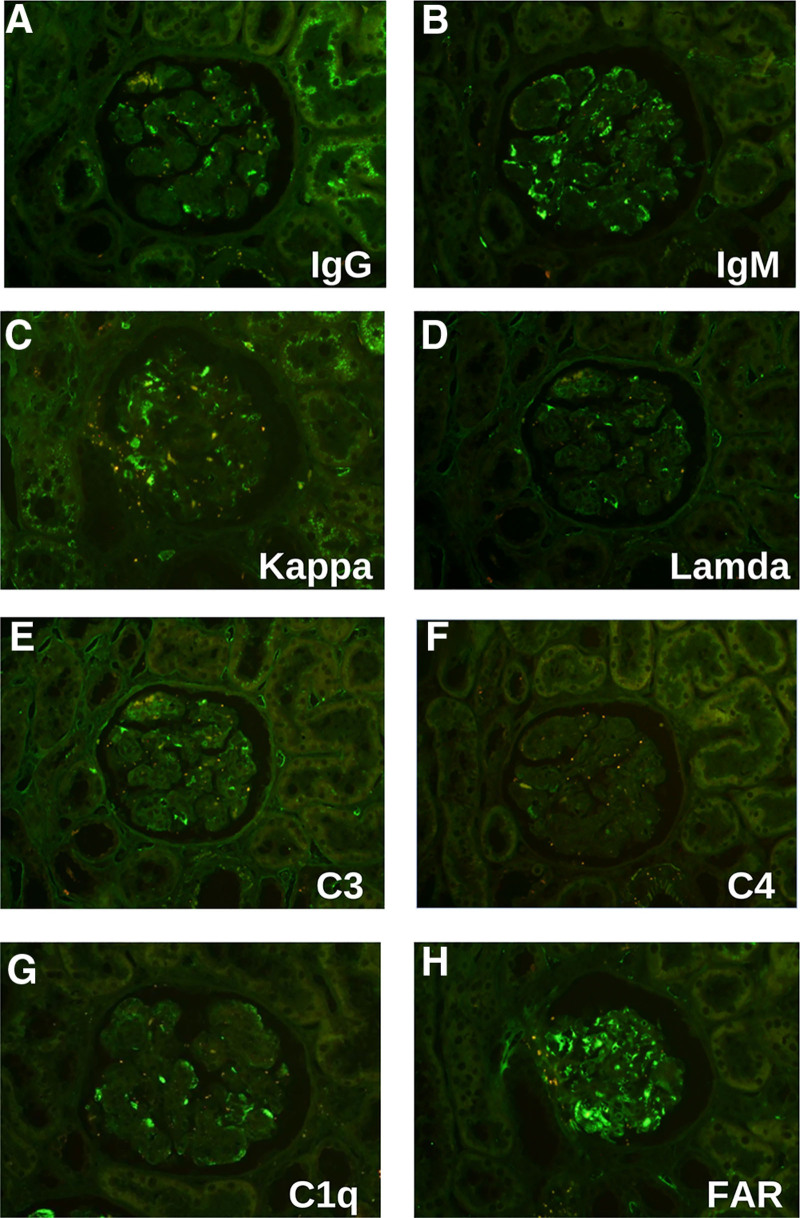
Immunofluorescence microscopy findings in 2022. Immunofluorescence analysis (original magnification, ×200) showing IgG (+, ++) (A), IgM (+, ++) (B), kappa (++) (C), lambda (+, −) (D), C3(+) (E), C4 (−) (F), Clq (+, ++) (G), and FAR (++, intracapillary companion) (H), with segmental capillary wall deposition. Ig = immunoglobulin.

**Figure 5.**
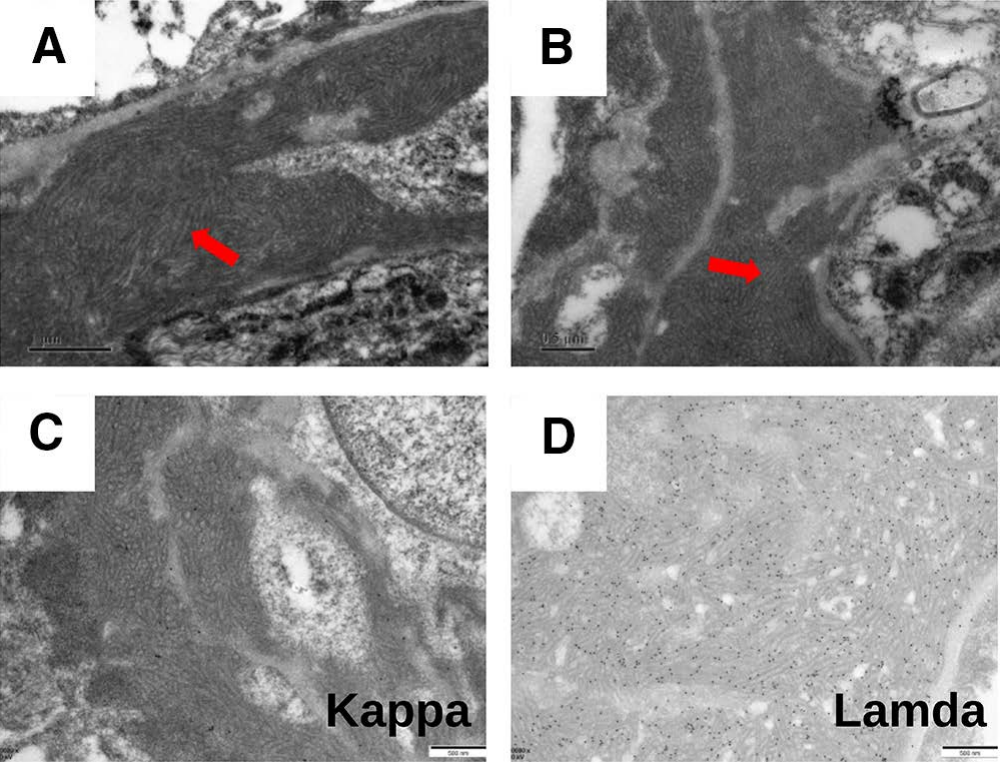
Electron microscopy findings. Electron microscopy showing a large number of microtubule-like structures in the subendothelial and thylakoid regions (diameter of around 60 nm) (A and B), with kappa (+, −) (C), and lambda (++) (D) (arrows). The images are from the Electron Microscopy Department of Peking University First Hospital.

**Figure 6.**
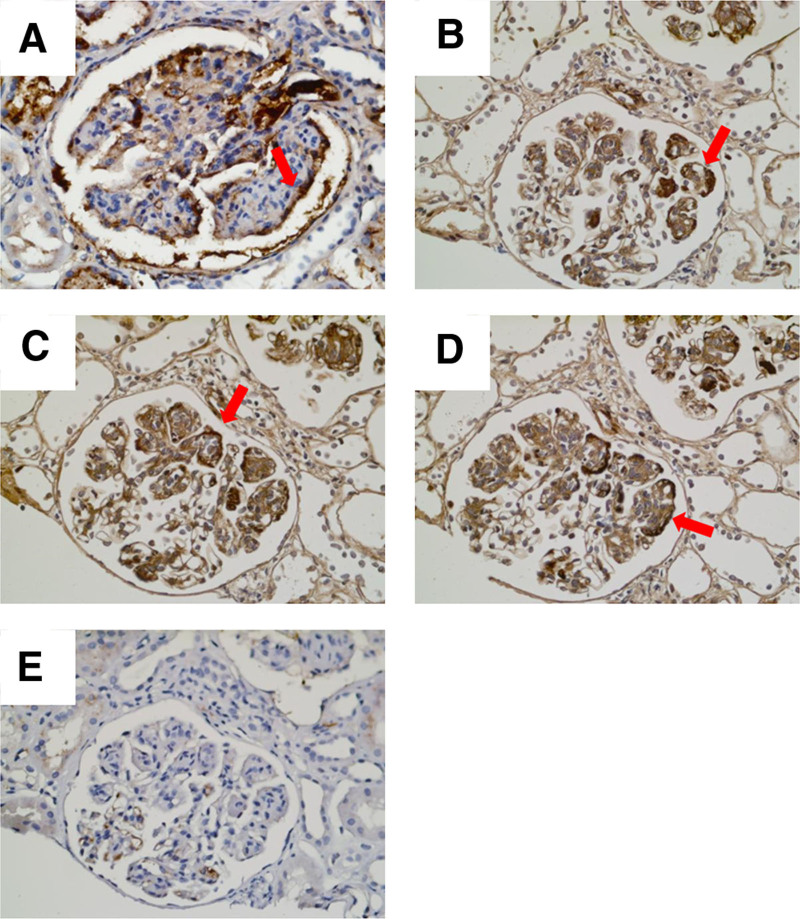
Immunohistochemistry study, original magnification, ×400. Immunohistochemistry showing positive granular staining along the glomerular basement membrane for IgG (A), IgG1 (B), IgG2 (C), IgG3 (D), and IgG4 (E) (arrows). The images are from the Electron Microscopy Department of Peking University First Hospital.

The patient developed proteinuria 5 months after kidney transplantation, with 24-hour urine protein quantification of 1.02 g. Transplanted kidney puncture biopsy was performed, and light microscopy showed 13 glomeruli, with diffuse mild-to-moderate hyperplasia of the glomerular thylakoid cells and thylakoid stroma. Moreover, there was severe aggravation of segmental hyperplasia, segmental endothelial cell swelling with obvious hyperplasia, and insertion of hyperplastic thylakoid stroma into the capillary walls. Segmental thickening of the glomerular basement membrane, segmental double-track sign formation, subendothelial complex hemoglobin deposition, and plasma stagnation were seen in some capillaries, and inflammatory cell stagnation was seen in some glomeruli. There was mild vacuolar granular degeneration of renal tubular epithelial cells, visible tubulitis, and no obvious tubular atrophy. Small foci of lymphoid and macrophage infiltration were present in the interstitium, and there was no significant fibrosis in the interstitium. Two small clear arterioles were visible, and the walls of the small arterioles did not show notable thickening or inflammatory cell infiltration (Fig. 3A–E). Paraffin immunofluorescence showed the following results with segmental capillary wall deposition: IgG (+, ++), IgM (+, ++), IgA (−), kappa (++), lambda (+, −), C3 (+), and C4 (−) (Fig. 4A–E). Electron microscopy showed moderate-to-severe hyperplasia with insertion of glomerular mesangial cells and mesangial stroma, formation of a large number of microtubule-like structures in the subendothelial and mesangial areas with a diameter of around 60 nm, segmental widening of the lax layer within the basement membrane, and fusion of the epithelial pedicles (Fig. 5). Renal tubular epithelial cells showed vacuolar-like degeneration with an increase in lysosomes and partial atrophy. There was renal interstitial lymphomonocytic infiltration with collagen fiber proliferation. Immunohistochemistry(original magnification × 400) showing positive granular staining along the glomerular basement membrane for IgG, IgG1, IgG2, IgG3, and IgG4. (Fig. 6A–E, arrows). Therefore, the patient was considered to have ITG recurrence.

**Figure 7.**
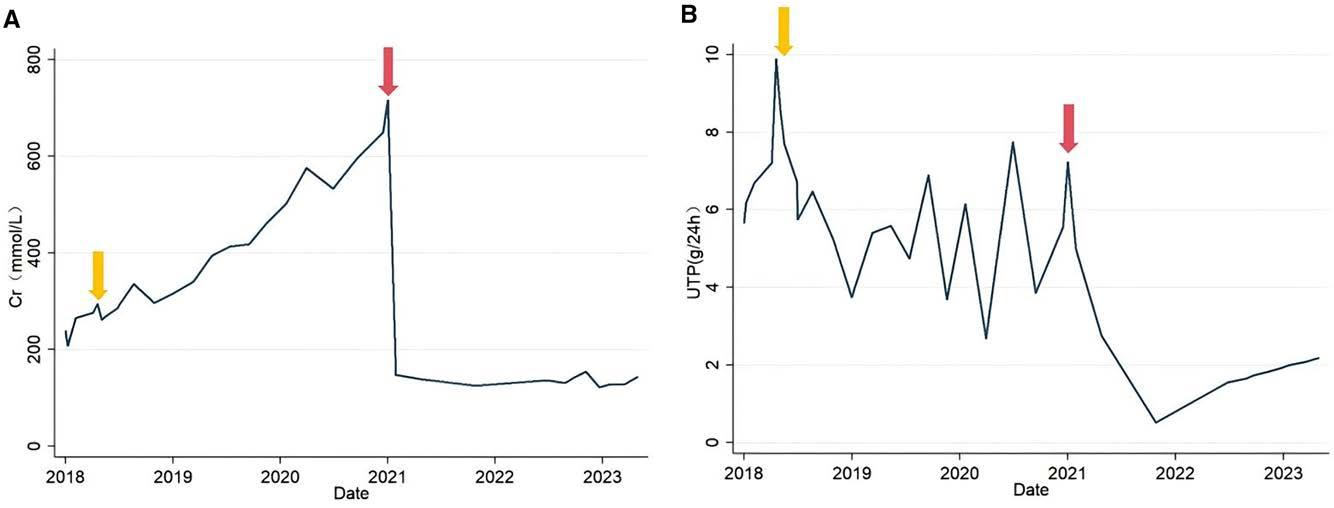
Serum creatinine (A) and urine total protein (B) versus time from rituximab treatment or kidney transplantation. The yellow arrows indicate the time at which rituximab was administered to the patient. The red arrows indicate when kidney transplantation surgery was performed.

After the patient had been diagnosed with ITG recurrence in the transplanted kidney, he was still regularly taking tacrolimus, and prednisone acetate. During the treatment period, we considered using rituximab again to deplete B cells. However, since the B-cell count remained consistently below 5 cells/μL, we concluded that B cell reconstitution had not occurred and that administering rituximab would be of limited clinical significance, so he was only given administered symptomatic treatment, and did not apply rituximab was not used again. During the follow-up period, the patient’s blood creatinine was stable (120–150 mmol/L) (Fig. 6A), but urine protein exhibited a gradual increase (Fig. 6B).

## 3. Discussion

The patient reported in the present study was initially admitted to the hospital for creatinine elevation and proteinuria, and he was diagnosed with ITG after combining the results of renal biopsy and excluding secondary factors. After the diagnosis of ITG, the patient was treated with rituximab, and blood creatinine stabilized at the initial stage of treatment. However, creatinine gradually increased in the middle and late stages of treatment, and renal function continued to deteriorate. Therefore, the patient underwent kidney transplantation, and he was admitted to the hospital again 5 months after kidney transplantation because of proteinuria. Renal biopsy showed recurrence of ITG, with immunofluorescence and electron microscopy suggesting restricted expression of kappa light chain, but no monoclonal Ig was detected in the blood or urine. The patient’s B-cell count was consistently < 5 cells/μL, so rituximab was not re-administered. Currently, the patient continues to take mizoribine, tacrolimus, and prednisone acetate His CD19-positive B-cell count, blood creatinine, and urine protein are being monitored, and his condition is stable.

ITG is a primary glomerulopathy with clinical manifestations similar to those of other primary glomerular diseases. Patients with ITG may present with proteinuria, hematuria, hypertension, and renal insufficiency. In Nasr et al.’s study, proteinuria was present in all patients (76% had proteinuria at nephropathic levels), and nephrotic syndrome was present in more than half of the patients (57%). The comorbidities of hypertension (88%), diabetes mellitus (15%), and autoimmune diseases (14%) were also present.^[3–5]^

The most common patterns of glomerular injury in patients with ITG are intracapillary proliferative glomerulonephritis, membranoproliferative glomerulonephritis, and membranous glomerulonephritis.^[2]^ Diagnosis relies on renal biopsy and electron microscopy. The key to the diagnosis of ITG is the presence of parallel, ordered microtubular structures with a diameter of 20 to 30 nm in the glomerulus that can be observed on electron microscopy. This feature often differentiates ITG from other glomerular disorders, such as cryoglobulinemia and fibrillary glomerulonephritis. In cryoglobulinemic glomerulonephritis, the deposits consist of short and curved microtubules or fibrous substructures with a diameter of < 30 nm.^[6]^ Fibrillary glomerulonephritis is characterized by a random arrangement of fibrous deposits with diameters of ≤ 30 nm, whereas ITG is mainly characterized by parallel fibrous deposits with diameters of > 30 nm. Moreover, patients with ITG tend to have hematological disorders, while patients with fibrillary glomerulonephritis have poor renal survival.^[7–9]^ In addition, these conditions can be differentiated by negative DNAJB9 staining in ITG patients.^[10]^

In the present case, a large number of microtubule-like structures were visible in the subendothelial and tunica albuginea areas, which were arranged in parallel and had a diameter of around 60 nm on electron microscopy. No abnormality was seen on immunofixation electrophoresis of the blood and urine, or on the blood tests measuring free light chains kappa and lambda. Therefore, the diagnosis of cryoglobulinemia, fibrillar glomerulonephritis, and monoclonal immunoglobulin deposits could be ruled out, and the diagnosis of ITG was considered to be clear.

The exact etiology of ITG is not clear. Some studies have suggested a hematological association. For instance, in Nasr et al.’s study, 6 of 16 patients were diagnosed with malignant hematological disorders, of which 3 had chronic lymphocytic leukemia, 2 had lymphoma, and 2 had multiple myeloma.^[3]^ Bridoux et al performed a fine ultrastructural analysis of lymphoma-associated ITG and found microtubular monoclonal IgG inclusion bodies in circulating and mesenchymal lymphoma cells, with the same ultrastructural manifestations of glomerular microtubular deposits as in ITG. These findings suggest that deposits in the microtubules in monoclonal ITG may be related to the structural and physicochemical properties of secreted IgM, which may be due to light chain amino acid substitutions in highly variable regions.^[11,12]^ In contrast, polyclonal ITG may be due to glomerular deposition of an unknown protein with the property of polymerizing into microtubules, which then triggers an autoimmune response.^[13,14]^ Therefore, patients with ITG should be evaluated for B-cell or plasma cell lymphoproliferative disorders, and they can be screened by serum and urine electrophoresis to detect monoclonal immunoglobulinopathies.

No specific drug treatments exist for ITG. No treatment has shown significant efficacy due to the lack of clinical randomized controlled trials. However, it has been shown that patients with underlying diseases, such as monoclonal gammopathy, malignancies, or autoimmune diseases, may benefit from B-cell or plasma cell-targeted therapies. A study by Javaugue et al explored the treatment and prognosis of 27 patients with ITG. In patients with hematological neoplasms, the use of targeted or empiric chemotherapy (e.g. cyclophosphamide, merti-methacolphenol ester) were effective for controlling disease progression, and in patients with monoclonal gammopathy of renal significance, the use of rituximab delayed the decline in renal function. For some patients with severe disease, plasma exchange or kidney transplantation may be required.^[15]^ A previous case report showed that a patient with ITG benefited from corticosteroids.^[16]^ In the present case, the patient lacked hematologic disease as an indication; therefore, there was no evidence for the use of targeted therapies.

Nasr et al proposed to categorize ITG into monoclonal and polyclonal types, with monoclonal ITG being considered present if immunofluorescence staining is predominantly IgG and restriction light chains and IgG subtypes are visible. Patients with monoclonal ITG have a higher incidence of lymphoma, multiple myeloma, and monoclonal gammaglobulinemia than those with polyclonal ITG. Monoclonal ITG is more commonly treated with clone-directed therapy, which results in a higher remission rate and a lower rate of ESRD, and it has a better prognosis than polyclonal ITG. This study demonstrates that clone-directed therapy can therapeutically alleviate monoclonal immunoglobulinopathy and improve proteinuria and renal function.^[17]^ As for monoclonal ITG, anti-B-cell therapy is often used as a first-line therapy. Rituximab has proven beneficial for the treatment of ITG, fibrillary glomerulonephritis, and monoclonal immunoglobulin deposits in previous studies.^[4,18–20]^ However, identifying and treating monoclonal gammopathy of renal significance requires a multidisciplinary approach involving nephrology, pathology, and hematology.

It has been shown that 50% of patients with ITG progress to ESRD within 5 years of diagnosis. Patients with ITG who progress to ESRD are eligible for dialysis or kidney transplantation, but disease recurrence can occur in the transplanted kidney.^[21,22]^ The pathology of recurrence is variable. For instance, in the study of Nasr et al., patients were found to have polyclonal ITG in the transplanted kidney on biopsy 10 months after transplantation. Owing to disease recurrence, second kidney transplantation was performed, and monoclonal ITG occurred after the second transplantation.^[4]^ In terms of prognosis, Table 2 presents case studies analogous to our case, focusing on patients who developed ITG in transplanted kidneys. It summarizes renal biopsy outcomes, treatments and variations in proteinuria and serum creatinine levels, offering valuable references for our study. Notably, 3 patients received rituximab therapy following recurrence, while other 3 patients were managed with alternative immunosuppressive treatment or no specific treatment. Patients treated with rituximab showed consistent improvement in proteinuria. In contrast, no significant reduction in proteinuria was observed in those receiving other therapies or no treatment. These findings may suggest a potential therapeutic efficacy of rituximab in managing ITG recurrence in renal transplant recipients.^[4,7,23,24]^ The present case was a posttransplantation ITG recurrence case. After recurrence, although the restricted expression of light chain kappa was found in the glomerular tissues, the patient’s B-cell count was persistently < 5 cells/μL, and the patient was not treated with rituximab again. The patient’s condition stabilized, and disease progression slowed. Figure 7 demonstrates the pretransplantation and posttransplantation trends in blood creatinine, urinary protein, and serum albumin. The changes suggest that the progression of ITG in the transplanted kidney is slower than that in the autologous kidney, which may be related to the long-term application of immunosuppressants after transplantation.

**Table 2:** Summary of previous reports evaluating ITG and its clinical characteristics, including the treatment plan for ITG recurrence after kidney transplantation.

References	Nasr et al (patient 1)	Nasr et al (patient 2)	Nasr et al (patient 3)	S. Sathyan	Xavier Carles	K. Venkateswara Rao
Age	56	46	57	50	30	59
Sex	Female	Male	Male	Female	Female	Male
Underlying conditions	CLL	None	None	MGUS	IgA lambda monoclonal gammopathy	–
Treatment of native disease	None	prednisone/cyclosporine	prednisone/rituximab	–	–_`_	–
Pattern of glomerular	MGN with	MGN	MPGN	–	–	Focal segmental
Injury in the native	Segmental	–	–	–	–	Necrotizing
Biopsy	Endocapillary proliferative features	–	–	–	–	Glomerulonephritis
Maintenance immunosuppressive regimen	Tacrolimus/pre dnisone/mycophenolate mofetil	First plan: cyclosporine/pre dnisone/mycophenolate mofetil Second plan: tacrolimus/prednisone/mycophenolate mofetil	Tacrolimus/prednisone/mycophenolate mofetil	Tacrolimus/mycophenolate mofetil/prednisone.	Prednisone/azathioprine/thymoglobulins/ cyclosporin A	Minnesota antilymphoblast globulin/azathioprine/so lumedrol/prednisone.
Serum creatinine at diagnosis of recurrent disease in mg/dL	1.0	2.0	4.98	1.9	3.2	2.8
24-h urine protein at diagnosis of recurrent disease in g/d	0.7	5.4	1.44	5.8	_-_	1.12
Treatment of recurrent disease	Fludarabine, cyclophosphamide and rituximab	Prednisone/cyclophosphamide/rituximab	None	The patient was given a trial of Rituximab 375 mg/m^2^ weekly for 4 wk.	Plasma exchanges/methylprednisolone/prednisolone	prednisone/solumedrol/a zathioprine
Serum creatinine at last follow up	1.5	3.2 at the second transplant	4.23	1.4	1.1	–
24-h urine protein at last follow up in g/d	0.2	3 at the second transplant	1.44	5	0.2	1.7

Some information was not available, which is indicated by “–”.

CLL = chronic lymphocytic leukemia, Ig = immunoglobulin, ITG = immunotactoid glomerulopathy, MGN = membranous glomerulonephritis, MGUS = monocloncal gammopathy of undetermined significance, MPGN = membranoproliferative glomerulonephritis.

## 4. Conclusions

The treatment and management of ITG faces multiple challenges, including disease recurrence, side effects of treatment, and the risk of renal failure. In terms of the limitations of this study, the present case lacked comprehensive testing related to the hematologic system; therefore, we failed to clarify the presence of hematologic pathology. Even though we consider that the recurrence in the renal allograft may be associated with the deposition of circulating immune complexes in the patient’s blood, the lack of a comprehensive assessment of the hematological and immunological profile makes it challenging to ascertain the underlying etiology of the recurrence. Consequently, this limitation hinders our ability to implement targeted therapeutic interventions. Secondly, the extended disease duration and prolonged follow-up period resulted in partial data gaps and statistical discontinuities, which may have compromised the observation of clinical outcomes. Thirdly, there is insufficient comparative analysis with similar cases for controlled study. Nevertheless, the findings of this study are useful because, owing to the small number of reported cases of ITG recurrence after kidney transplantation of ESRD, there is no clear treatment plan for this population. Therefore, more clinical cases are needed to explore the treatment options and prognosis of ITG in these patients, and this study adds to the existing literature on this topic.

## Acknowledgments

We appreciate the Electron Microscopy Department of Peking University First Hospital for its technical assistance.

## Author contributions

**Investigation:** Shuxin Yu, Fanqian Cheng.

**Methodology:** Fanqian Cheng.

**Resources:** Jinyu Yu, Shan Wu, Weixia Sun.

**Writing – original draft:** Shuxin Yu, Xiaoxuan Zhao.

**Writing – review & editing:** Shuxin Yu, Chunbo Zhao, Shan Wu.
